# Digital impression making in dentistry: A review

**DOI:** 10.6026/973206300213283

**Published:** 2025-09-30

**Authors:** Anjali Sharma, Ameer Akhil Ahmed Shaik, Sumit Bhatt, Aishwarya Handa, Sonali Perti, Deepashree C., Pratik Surana

**Affiliations:** 1 Department of Conservative Dentistry and Endodontics, Teerthanker Mahaveer Dental College & Research Centre, TMU, Moradabad, Uttar Pradesh, India; 2 Department of Dental Surgery and General Dentistry, Narayana Dental College, Nellore, India; 3 Department of Oral and Maxillofacial Surgery, Rajasthan Dental College & Hospital, Nirwan University, Jaipur, Rajasthan, India; 4 Department of Conservative Dentistry & Endodontics, Bharati Vidyapeeth Deemed to be University Dental College and Hospital, Pune, Maharashtra, India; 5 Department of Prosthodontics, Kalinga Institute of Dental Sciences, KIIT University, Campus 15, Patia, Bhubaneswar, Odisha, India; 6 Department of Prosthodontics and Crown and Bridge, V S Dental College and Hospital, Bengaluru, Karnataka, India; 7 Department of Pedodontics and Preventive Dentistry, Maitri College of Dentistry and Research Centre, Durg, Chhattisgarh, India

**Keywords:** Digital impression, conventional impression, dentistry

## Abstract

The adoption and impact of digital impression-making in dentistry is of interest. It highlights key benefits such as enhanced accuracy, reduced patient discomfort, faster restoration times and improved collaboration among dental professionals. Thus, data shows the transformative role of digital technology in enhancing patient care and dental practice efficiency.

## Background:

The incorporation of digital impressions in the creation of crowns, bridges and dentures has truly transformed prosthodontics, allowing for a more efficient and precise way to capture intraoral data [1]. While the idea of digital impressions emerged in the late 1980s, it wasn't until the initial 2000s that the technology gained traction and became commercially viable. Early digital scanners were large and costly, making them accessible only to select innovative dental practices. However, thanks to technological advancements and the arrival of compact, reasonable scanners, digital imaging systems have become commonplace in dental clinics around the globe [2, 3]. Digital impressions offer several advantages, including greater precision and specificity in contrast to conventional methods, less uneasiness by eliminating messy materials that can trigger a gag reflex and quicker turnaround times for restorations since these impressions can be sent immediately to labs [4]. Furthermore, digital models aid in patient education and treatment planning, allowing for better visualization and manipulation of dental concepts. Overall, digital dentistry brings several upsides and progressions to the field, revolutionizing the way dental professionals operate. This method results in more streamlined and successful treatments, better communication, reduced costs and ultimately improves the overall experience and outcomes for patients [1, 5]. Therefore, it is of interest to provide an in-depth discussion of digital impressions in dentistry.

## Impression from past to present:

Impression making in dentistry is a quite recent development. In 1856, Dr. Charles Stent advanced the use of gutta percha for correcting oral deformities, marking a shift from traditional materials like beeswax. The late 1950s introduced polysulfide rubber, improving upon hydrocolloids. In 1965, ESPE, GmbH launched Impregum, first elastomeric impression material specifically for dentistry, offering fast setting, excellent flow, high detail reproduction, adequate tear strength and low shrinkage [6]. After condensation silicone impression materials were introduced, they faced challenges with dimensional accuracy. However, the advent of addition silicone vinyl polysiloxane impression materials addressed these dimensional issues, along with unpleasant taste and odor and a high modulus of elasticity. These new materials provided outstanding tear strength, excellent flow characteristics and maintained their shape without distortion, even when models were not poured promptly [4, 7]. Besides the inherent accuracy issues with elastomeric materials, additional distortions can arise from errors in mixing, impression techniques, the use of flexible trays and transferring impressions to the dental lab. Humidity control in the lab is also essential to ensure accurate setting of gypsum models. Fortunately, newer technologies, like digital scanners for impression making, are a significant advancement. Three-dimensional (3D) digitizing scanners have been utilized in dentistry for over 20 years and continue to evolve, capturing virtual impressions more effectively. Today's CAD/CAM dental systems can directly integrate data from precise digital scans into milling machines, allowing for the creation of restorations from ceramic or composite resin blocks without the need for physical impressions [4, 8].

## The functionality of digital impression (3D Imaging):

Intraoral scanners operate through a sophisticated blend of optics, image sensors and software systems, enabling them to create accurate and comprehensive scans of the oral cavity [3].

## Optical components:

These scanners employ cutting-edge optical elements-like lenses and mirrors-to obtain high quality intra oral images. These components are meticulously engineered to concentrate on the intricate surface specifics of teeth and soft tissues, ensuring precise capture of even the finest features [9, 10].

## Light projection technologies:

Numerous intraoral scanners make use of structured light or confocal imaging techniques, projecting light patterns onto the surfaces they examine. By studying changes in these patterns as they interact with dental structures, scanner can generate highly accurate 3D surface maps [9, 10].

## Immediate feedback and image capture:

Intraoral scanners feature a mechanism that offers real-time feedback to the operator, commonly displayed as live images on a computer monitor. As the user navigates the scanner through oral cavity, the device continuously captures and processes images, delivering instant visual feedback to guarantee thorough coverage and high data quality [3, 9 and 10].

## Multi-axis capability:

These scanners are equipped with a flexible head or tip that allows for movement in multiple axes, providing users the ability to reach and scan every area of the oral cavity. This adaptability is crucial for effectively capturing the entire dental arch, including those hard-to-reach posterior regions that traditional impression methods struggle with [3].

## Software algorithms and data analysis:

The 3D data collected by intraoral scanner is processed through advanced software processes that seamlessly combine individual images to form a cohesive digital model. These systems are engineered to address issues like motion artifacts, reflection interference and occlusal inconsistencies, ensuring a precise depiction of the dental structure [3].

## Wireless and compact design:

Contemporary intraoral scanners frequently incorporate wireless connectivity and lightweight designs, providing enhanced mobility during the scanning process [3].

## Advantages and disadvantages of conventional impressions [1, 2 and 11]:

## Advantages:

[1] Widely recognized and trusted method.

[2] Requires minimal equipment.

[3] Affordably priced, generally low to moderate.

[4] Proven accuracy.

[5] Clinical procedure is straightforward and reliable.

## Disadvantages:

[1] Can be quite messy.

[2] May cause discomfort for patients.

[3] Air bubbles or debris can lead to mistakes.

[4] Necessitates keeping materials and trays in stock.

## Advantages and disadvantages of digital impressions [1, 2, 11 and 12]:

## Advantages:

[1] Comparable accuracy to conventional impressions.

[2] User-friendly after a brief learning period.

[3] No mess involved.

[4] Reduced discomfort for patients.

[5] Eliminates the need for stocking materials and trays.

[6] Removes the necessity for disinfection.

[7] No risk of cross-contamination.

[8] Simplifies transfer to the laboratory.

[9] Eliminates the need to articulate casts.

[10] Removes tasks of pouring impressions, creating bases and trimming.

[11] Allows for long-term data storage.

## Disadvantages:

[1] Dentists may be unfamiliar with the technology.

[2] Requires complex digital equipment.

[3] Higher initial purchasing cost.

## Conventional versus digital impression:

Table 1 highlights the compression of both conventional and digital impression [1, 3 and 11].

## Discussion:

As defined in the Glossary of Prosthodontic Terms, an impression is "a reverse copy of an object's surface; essentially a mold of the teeth and surrounding structures used in dentistry." Taking an impression is a crucial step in creating fixed prosthetic restorations like crowns, bridges, inlays, on lays and implants, as well as removable dentures, a practice that has been fundamental in dentistry for many years [13]. While conventional methods dominated the field for a long time, today, elastomeric impression materials, particularly polyvinyl siloxane and polyether, are trusted for their exceptional accuracy in capturing these impressions [13, 14]. In the early 1980s, digital impression systems began to take shape when Werner Mörmann envisioned a way to streamline the treatment process. He discussed this concept with his friend, electronic engineer Marco Brandestini and leading to the development of digital impression tools featuring optical reading systems. Both digital and conventional impression methods present their own advantages and disadvantages. Conventional methods often involve multiple steps, increasing the chances of errors. In contrast, the standardized milling stage in digital impressions, along with fewer steps, minimizes the risk of mistakes and enhances adaptability. As a result, digital methods are generally favored for their time efficiency and clinician preference [13, 15].

## Assessing the precision and trustworthiness of digital impressions in comparison to traditional impressions:

Ender and co-workers evaluated the accuracy and reliability of full-arch dental impressions using both traditional and digital techniques. They tested four digital technologies and traditional materials. A highly accurate reference scanner compared the precision of conventional and digital impressions of the same tooth morphology. Outcome showed that CEREC Bluecam, vinyl siloxanether and direct scannable vinyl siloxanether had upmost accuracy, with digital technologies providing significantly greater regional detail than traditional methods [16]. According to Giachetti, traditional impressions created with high-precision materials tend to be more accurate than their digital counterparts [17]. However, Tabesh [18] and Hasanzade [19] found that restorations made using a digital workflow achieved better marginal fit accuracy than those made with conventional impressions. Marques S *et al.* conducted a review that identified 108 articles, with 21 meeting the inclusion criteria. The studies focused on five main areas: the accuracy of digital impressions in implant dentistry, the design and material of intraoral scan bodies, scanning techniques, the impact of implant depth and angulation on accuracy, and the performance of different intraoral scanners. Key factors influencing the accuracy of digital impressions include implant depth, operator experience, scanner type, and environmental conditions. Notably, the design and material of intraoral scan bodies, along with the scanning technique, significantly affect the trueness and precision of digital impressions in implant dentistry [20].

## Impression timing:

When it comes to time efficiency, some discrepancies were noted among the studies; however, it seems clear that intraoral scanning (IOS) impressions generally require less time than traditional impressions. IOS has been associated with a shorter average repetition time but a higher average repetition count. This is largely because doctors find it easier to rescan specific areas that may have been missed, whereas with traditional impressions, any error necessitates restarting the entire process. A significant benefit of digital impressions is the ability to rescan and preview missing sections, facilitating real-time feedback-something that conventional impressions lack, as errors often go unnoticed until the impression material has set or a plaster model has been created. Researchers such as De Oliveira *et al.* [21] and Manicone *et al.* agree that the impression-taking phase is quicker with digital methods compared to conventional techniques [22].

## Cost-efficiency of digital impressions:

While the upfront costs of transitioning to digital impressions may seem high, they can ultimately result in substantial long-term savings for dental practices. This is largely due to the elimination of expensive traditional impression materials, such as alginate or polyvinyl siloxane, which need to be replenished frequently. Furthermore, digital impressions streamline the process by enabling easy digital file sharing with dental labs for restoration fabrication, reducing the number of necessary patient visits and eliminating the hassle of shipping physical models back and forth. This approach not only reduces time and shipping expenses but also minimizes the chances of damage during delivery. Plus, digital impressions offer a more accurate depiction of a patient's dental anatomy, leading to restorations that fit better and greater patient satisfaction [10].

## Patient comfort:

Patients often view digital impressions as a faster and more convenient choice, resulting in a better overall experience. Embracing digital impressions can significantly enhance the dental journey for both practitioners and patients. Hung Lam and his colleagues found that the digital impression method is a strong alternative to traditional rubber impressions. This technique not only reduces the time needed for both the patient and the dentist but also improves patient comfort, making for a more pleasant treatment experience [23].

## Scanning protocol:

Giménez-González *et al.* have highlighted that the scanning protocol used can greatly affect the precision of digital impressions. Their research points out that as one moves along the arch, both distance and angle discrepancies tend to increase, underscoring the necessity of initiating the scanning process at the restoration site for partial restorations to ensure maximum accuracy. Additionally, the design of the intra-oral scan body (ISB) plays a vital role, as its visibility can directly influence the precision of digital impressions. They advise using longer ISB designs for implants that are placed deeper, suggesting that the depth of the implant may also affect impression accuracy. Although several researchers have looked into how ISB design impacts digital impression accuracy, there is still no clear agreement on which design yields the best outcomes [24].

## Application of digital impression in various branches of dentistry:

Digital impressions, made possible by intraoral scanners, are transforming restorative dentistry by significantly improving precision and planning. These advanced tools facilitate the creation of digital wax-ups, empowering patients to collaborate on personalized designs that align with their dental goals. Additionally, digital technology simplifies the processes for mock-ups and surgical guides, making planning quicker and more efficient than traditional methods. This integration not only fosters better treatment outcomes but also enhances patient satisfaction within dental practices. In pedodontics and orthodontics, digital impressions further enhance treatment planning and monitoring, allowing for the customization of braces and aligners tailored to individual growth patterns. Furthermore, early diagnosis of dental wear is critical for effective treatment. Research indicates that intraoral scanners excel at quantifying the progression of dental wear, demonstrating higher sensitivity in detecting changes compared to specificity. This capability not only improves diagnostics but also fosters greater awareness among patients about their oral health. As technology evolves, the role of digital impressions in restorative dentistry continues to expand, paving the way for a brighter, more efficient future in dental care. The integration of intraoral scanners into dental practices represents a significant leap forward, ensuring that patients receive the best possible care across all facets of dentistry [25].

## Limitation of digital impression:

Digital impressions face challenges like limited accessibility in certain regions due to infrastructural constraints and financial limitations of dental practices. Many offices struggle to afford the costs of implementing and maintaining these systems, which restricts technology availability. However, initiatives are underway to enhance accessibility, including training and support for interested practices and financing options. As these efforts progress, more dental experts will have the opportunity to benefit from digital impressions, improving patient care and efficiency.

## Future prospective:

The future of digital impression making in dentistry looks promising, with advancements in technology enhancing accuracy, efficiency and patient comfort. 3D scanning and imaging techniques provide precise digital models, reducing the need for traditional impressions. This shift minimizes errors, streamlines workflow and enables faster treatment planning. Integration with CAD/CAM systems will further revolutionize restorative procedures, leading to improved patient outcomes and personalized dental care.

## Conclusion:

Digital impression-making significantly enhances dental practice by increasing accuracy, efficiency and patient satisfaction. While challenges such as accessibility and financial constraints exist, on-going technological advancements and support initiatives are paving the way for broader implementation. As adoption grows, digital impressions are set to play a crucial role in improving overall treatment outcomes and the future of dentistry.
